# Ultrasonography in pediatric inflammatory bowel diseases

**DOI:** 10.1186/1824-7288-40-S1-A29

**Published:** 2014-08-11

**Authors:** Mauro Massimetti, Francesca Mangianti, Rino Agostiniani

**Affiliations:** 1 Pediatric Department, S. Jacopo Hospital, Pistoia 51100, Italy

## 

Inflammatory bowel diseases (IBD) are an heterogeneous group of chronic disorders of intestinal inflammation characterised by periods of remission and exacerbation. Crohn’s disease (CD) an ulcerative colitis (UC) are the two major clinical subtypes of IBD [1]. CD is caracterised by transmural inflammation in a non contiguos pattern anywhere from the mouth to the anus. Ileocolonic region is the most common location of disease in pediatric CD. Classically, UC involves disease that extends proximally for a variable distance from the rectus, with involvement of the superficial layers of the colonic mucosa. Pancolitis is the most frequent presentation of UC in childhood. Disease courses are different not only in childhood from adult life but olso in the different ages of pediatric patients [2]. Definitive diagnosis of IBD relies on endoscopic and histologic findings often supported by radiologic imaging. Ultrasound scanning as innocuos and ubiquitary imaging tecnique can be used both as screening diagnostic tool in patients with suspected IBD than in the clinical management of patients with proven IBD in the effort of detect extension, grade of activity and early individuation of complications in the follow-up. Ultrasound can be performed as a standard examination without preparation called transaddominal ultrasonography (TUS) or associated with previous ingestion of an oral contrast solution that produce an osmotic fluid distension of intestinal lumen for a more sensitrive and detailed valutation of sonographic aspects of the bowel wall called small intestine contrast ultrasonography (SICUS). In Crohn disease recent data in literature show that SICUS improves sensitivity in detecting small bowel lesions both in previous undiagnosed patients from 75% to 100% than in patients with proven CD from 76% to 100% compared to TUS [3]. The execution of ultrasound in the evaluation of pediatric intestinal tract requires time, good tecnique and experience of the sonographer. The main goal of this presentation is to show the most important tecnical aspects of the execution of ultrasound examination in pediatric IBD.
